# Vitamin supplementation and its effect on incident type 1 diabetes mellitus and islet autoimmunity: a systematic review and meta-analysis

**DOI:** 10.3389/fimmu.2025.1505324

**Published:** 2025-04-09

**Authors:** Chen Ee Low, Nicole Shi Min Chew, Sounak Rana, Sean Loke, Run Ting Chin, Shih Ling Kao, Ainsley Ryan Yan Bin Lee, Sen Hee Tay

**Affiliations:** ^1^ Yong Loo Lin School of Medicine, National University of Singapore, Singapore, Singapore; ^2^ Division of Endocrinology, Department of Medicine, National University Hospital, Singapore, Singapore; ^3^ Division of Rheumatology and Allergy, Department of Medicine, National University Hospital, Singapore, Singapore

**Keywords:** type 1 diabetes mellitus, islet autoimmunity, vitamin supplementation, vitamin D, vitamin B

## Abstract

**Introduction:**

The prevalence of type 1 diabetes mellitus (T1DM) is set to rise annually with long-term implications on the quality-of-life. Supplementation with vitamins has garnered interest in recent years due to its association with the development of islet autoimmunity (IA) and T1DM. This systematic review aims to investigate the relationship between vitamins supplementation on the development of IA or T1DM or progression of IA to T1DM.

**Methods:**

This PRISMA-adherent systematic review involved a systematic search of PubMed, Embase and Cochrane for all studies that evaluated the odds (pre-calculated pooled OR) and risk (RR) of IA, T1DM, or progression of IA to T1DM after supplementation with vitamins. Random effects meta-analyses were used for primary analysis.

**Results:**

15 studies were included. Meta-analyses observed that vitamin D did not modify the odds of developing T1DM (Pooled OR=0.55, 95%CI: 0.22-1.38) or IA (Pooled OR=0.91, 95%CI: 0.67-1.25). The relative risk of developing T1DM was almost significant (RR=0.66, 95%CI: 0.41-1.06), emphasizing the need to conduct further large-scale cohort studies. Systematic review revealed that vitamin B supplementation did not influence the risk of T1DM and progression of IA to T1DM. Additionally, there was an association between higher maternal education levels and higher levels of vitamin D supplementation in their offspring.

**Conclusion:**

In conclusion, we found no significant benefit with the use of various vitamins in modifying the risk of developing IA, T1DM or progression of IA to T1DM. Our study provides a foundation for future research by contributing to the evolving landscape of nutritional immunology.

**Systematic review registration:**

https://www.crd.york.ac.uk/prospero/, identifier CRD42024540524.

## Introduction

Type 1 diabetes mellitus (T1DM) is characterized by high blood sugar levels secondary to low blood insulin levels due to the autoimmune destruction of pancreatic beta islet cells (1). T1DM is commonly preceded by a pre-clinical phase of islet autoimmunity (IA), defined by the presence of circulating glutamic acid decarboxylase, insulin and protein tyrosine phosphatase-2 autoantibodies. Individuals with IA are at high risk of progression to T1DM (2).

The prevalence of T1DM is set to rise 0.34% annually, with an estimated 8.4 million people living with the condition in 2021, and a projected 13.5-17.4 million in 2040 (3). Long-term complications include diabetic retinopathy, neuropathy and nephropathy, the avoidance of which entails rigorous blood sugar monitoring and control (4). Individuals with type 1 diabetes mellitus experience a lower health-related quality-of-life (5) and psychological distress (6). Beyond the individual level, the cost to public health imposed by the condition is onerous. Individuals with type 1 diabetes mellitus, although fewer in number, have significantly higher mean total medical costs per patient per year compared with individuals with type 2 diabetes mellitus (7). Therefore, there is merit in investigating factors that may be protective against the development of T1DM, the development of IA, and the progression of IA to T1DM.

Vitamin supplementation has been studied in association with the development of IA and T1DM (8). Carakushansky et al. demonstrated a significantly high prevalence of vitamin D insufficiency in a cohort of children with T1DM (9). Similarly, Koshy et al. reported a 45% prevalence of vitamin B12 deficiency in a cohort of individuals with T1DM. T1DM develops at an early age, with the first peak in incidence at 4-7 years old. Therefore, early intervention is of particular interest to preventing its occurrence. *In vitro*, vitamin D acts as an immunosuppressant to reduce lymphocyte proliferation and cytokine production which are components of IA seen in T1DM (10). Other vitamins have also been investigated in association with T1DM and IA (11, 12). For example, niacin, vitamin B12 and riboflavin are components of a dietary pattern linked to a metabolomic profile negatively associated with the progression of T1DM (13) and have been demonstrated to reduce progression to T1DM (11). A systematic review of the effect of vitamin supplementation on the prevalence and progression of IA to T1DM may help provide evidence-based guidance for mothers on supplementing with antenatal and postnatal vitamins. This systematic review aims to investigate the relationship between vitamin supplementation on the development of IA, T1DM or progression of IA to T1DM. Secondary outcomes include evaluating the prognostic factors affecting maternal usage of vitamin supplementation.

## Methods

The systematic review was reported according to the Preferred Reporting Items for Systematic Reviews and Meta-Analyses (PRISMA) guidelines. Our protocol is registered on PROSPERO (CRD42024540524).

### Search strategy

Literature search was performed in PubMed, Embase, and Cochrane. Our search strategy combined terms for vitamins, T1DM and IA. We used the database-controlled vocabulary to search subject headings. Appropriate truncations from a spectrum of synonyms were used to search title and abstract keywords. The search strategy was translated between the databases. Examples of the search strategies for PubMed and EMBASE are found in **Supplementary Table 1**.

### Inclusion and exclusion criteria

**Figure 1:**
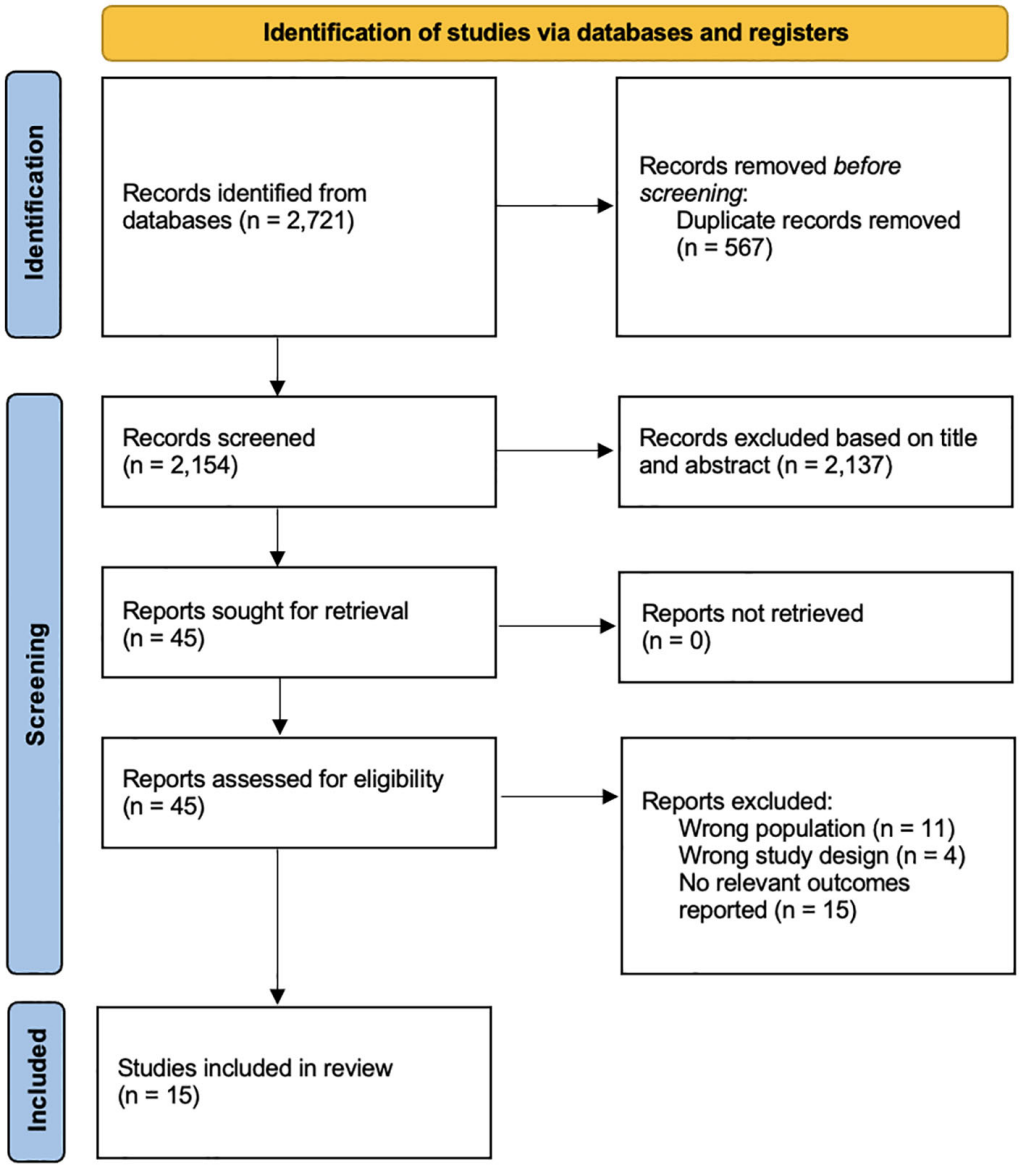
PRISMA flowchart.

Two reviewers independently screened titles and abstracts of the studies for eligibility. Any discrepancies were independently assessed by a third reviewer. All peer-reviewed English-language studies published inception to 27 September 2024 that evaluated the risk of IA, T1DM, or progression of IA to T1DM following the supplementation of vitamins were included. Due to the limited number of studies, all studies were included regardless of age to ensure adequate representation of the current literature. Non-empirical studies, grey literature, abstracts and studies on maternal supplementation were excluded. Grey literature searched include pre-prints and theses. The selection process is shown in **Figure 1**.

### Data extraction

Two reviewers independently performed the extraction. Subject matter information included the aim of the study, demographics, and characteristics of control group, and main findings of the study. The effect of vitamin D supplementation on the outcomes were quantified by pooling the precalculated odds ratio (OR) presented by the individual studies and calculating relative risk ratios (RR) from the absolute number at risk and events of both the exposed and control groups. We extracted the pooled OR, the 95% confidence interval (95%CI) and the absolute number at risk and events of both the exposed and control groups. We calculated the standard error of the pooled OR, according to Cochrane guidelines (14). Outcomes with insufficient data for meta-analysis were pooled using a synthesis without a meta-analysis approach.

### Statistical analysis

All analyses were performed on R (version 4.1.0) using the *meta* and *metafor* packages. A two-sided P value of <0.05 was considered as statistically significant. Studies were pooled for meta-analysis using ORs and RRs. Sensitivity analysis for publication bias was conducted using the leave-one-out and outlier analysis. Between-study heterogeneity was represented using I2 and τ2 statistics. I2 of less than 30% showed low heterogeneity between studies, 30% to 60% demonstrated moderate heterogeneity, and more than 60% revealed substantial heterogeneity (15).

### Risk of bias

Two independent reviewers assessed for the risk of bias of the included studies using the Joanna Brigg’s Institute (JBI) Critical Appraisal tool (16). Discrepancies were resolved by a third reviewer.

## Results

15 studies were included from 2,721 records (**Figure 1**) (8, 11, 12, 17–28). 2,704 records were excluded after removing irrelevant studies with the wrong population, design, outcomes and duplicates. Out of the 15 studies, four were from the United States (11, 18, 22, 28), three from Finland (8, 19, 29), two from Europe (23, 27), one each from Sweden (25), Denmark (30), Egypt (26), Algeria (24), Norway (12) and Italy (17). Nine studies were cohorts (11, 18–21, 25, 27, 28, 31), five were case-controls (12, 17, 22, 24, 26) and one was a randomized controlled trial (23). 386,116 total participants were studied. The overall characteristics of the studies can be found in **Table 1**.

**Table 1:** Characteristics of the included studies stratified by outcomes of interest (type 1 diabetes mellitus, islet autoimmunity and progression of islet autoimmunity to type 1 diabetes mellitus).

Author	Publication year	Region of study	Study design	Gender male (%)	Mean age (SD)	Control Characteristics	Total number of participants	Exposures	Main exposure
Type 1 Diabetes Mellitus									
Awadalla	2017	Egypt	Case-control	0.49	7.94 (3.39)	Age and sex matched, healthy controls	408	1. Vitamin D 2. Breast-feeding 3. Sun	Vitamin D
Brikhou	2023	Algeria	Case-control	0.46	NR	Non-diabetic	335	1. Vitamin D 2. Sun exposure 3. Diet	Vitamin D
Gorham	2012	USA	Case-control	NR	NR	Age and sex matched, healthy controls	2000	1. Vitamin D	Vitamin D
Jacobsen	2015	Denmark	Cohort	0.51	NR	–	331623	1. Vitamin D	Vitamin D
Hakola	2024	USA	Cohort	0.51	NR	–	8500	1. Vitamin D 2. Vitamin B	Vitamin D
Elhassan	2023	USA	Cohort	0.49	NR	–	2547	1. Multivitamin	Multivitamin
Hypponen	2001	Finland	Cohort	0.51	NR	–	12055	1. Vitamin D 2. Rickets	Vitamin D
Lund-Blix	2015	Norway	Cohort	0.51	NR	–	908	1. Vitamin D 2. Breastfeeding 3. Diet	Breastfeeding
Stene	2003	Norway	Case-control	0.51	Cases: 10.9 (3.4), Controls: 9.3 (4.1)	–	545	1. Cod liver oil 2. Other vitamin D supplements	Cod liver oil
Gale	2004	Europe	RCT	0.53	NR	–	552	1. Nicotinamide	Nicotinamide
Tenconi	2007	Italy	Case-control	0.55	Cases: 23.29 (9.36), Controls: 23.38 (9.64)	Age and gender matched, healthy controls	477	1. Diseases 2. Vitamin D 3. Bottle feeding	Vitamin D
EURODIAB	1999	Europe	Case-control	NR	NR	Healthy	3155	1. Vitamin D	Vitamin D
Marjamaki	2010	Finland	Cohort	NR	NR	–	4297	1. Vitamin D	Vitamin D
Islet Autoimmunity									
Simpson	2011	USA	Cohort	0.52	NR	–	2644	1. Vitamin D	Vitamin D
Hakola	2024	USA	Cohort	0.51	NR	–	8500	1. Vitamin D 2. Vitamin B	Vitamin D
Lund-Blix	2015	Norway	Cohort	0.51	NR	–	908	1. Vitamin D 2. Breastfeeding 3. Diet	Breastfeeding
Brekke	2007	Sweden	Cohort	NR	NR	–	16070	1. AD drops 2. Vitamin D supplements	Vitamin D
Marjamaki	2010	Finland	Cohort	NR	NR	–	4297	1. Vitamin D	Vitamin D
Progress of Islet Autoimmunity to Type 1 Diabetes Mellitus									
Hakola	2024	USA	Cohort	0.51	NR	–	8500	1. Vitamin D 2. Vitamin B	Vitamin D
Elhassan	2023	USA	Cohort	0.49	NR	–	2547	1. Multivitamin	Multivitamin
Simpson	2011	USA	Cohort	0.52	NR	–	2644	1. Vitamin D	Vitamin D

NR, Not reported; RCT, Randomized Controlled Trial; SD, standard deviation.

### Developing T1DM

**Figure 2:**
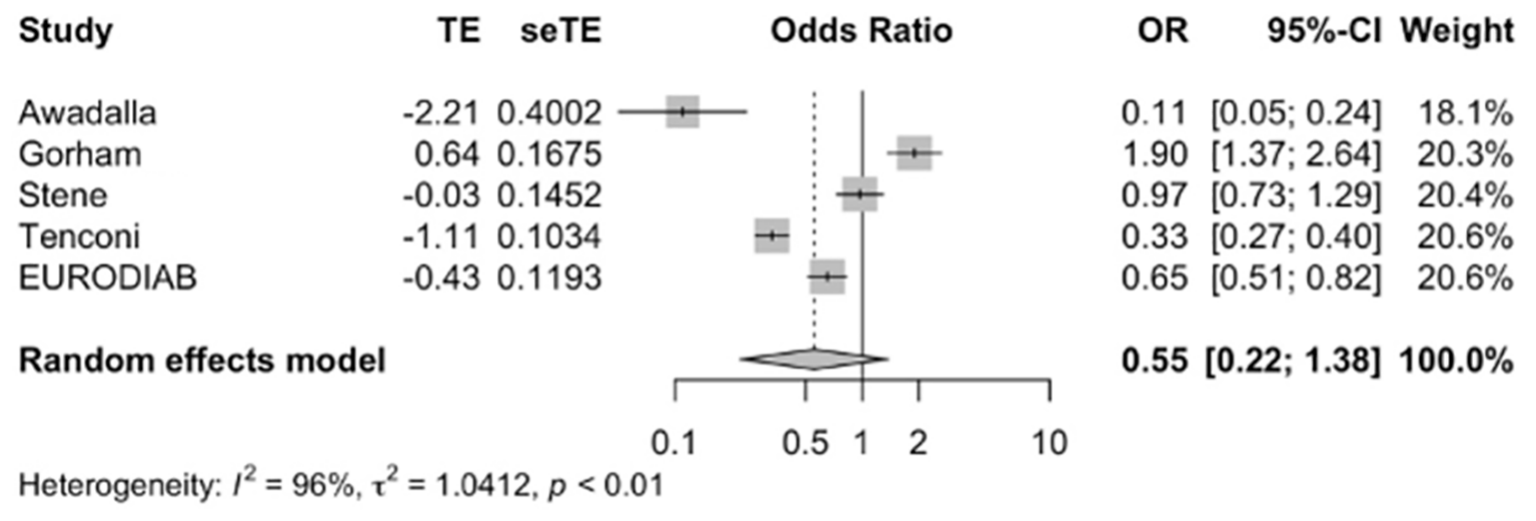
Precalculated pooled odds ratio of vitamin D supplementation on T1DM. TE, estimate of treatment effect; seTE, standard error of treatment estimate; OR, odds ratio; CI, confidence interval.

Meta-analysis of the five studies (12, 17, 22, 26, 27) (**Figure 2**) indicated that vitamin D supplementation does not seem to significantly affect the odds of developing T1DM (Pooled OR=0.55, 95%CI: 0.22-1.38). Out of the five studies, three studies individually demonstrated the protective effect of vitamin D supplementation (17, 26, 27). Overall, the five studies varied in study design, population, characteristics, timing of supplementation. The duration of supplementation differed across studies; Stene et al. and EURODIAB focused on early infancy while the rest focused on supplementation over longer periods (2, 27). Gorham et al. was the only study to show an increased risk of developing T1DM with vitamin D supplementation likely due to its unique focus on military members and their inclusion of dietary vitamin D intake alongside pure supplementation (22).

**Figure 3:**
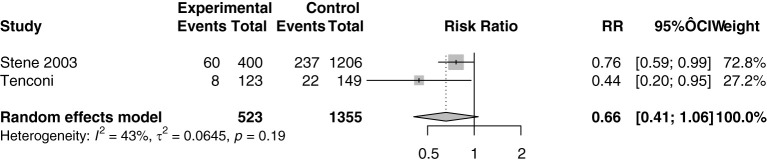
Relative risk ratio of vitamin D supplementation and T1DM calculated from the absolute number at risk and events of the exposed and control group. RR, risk ratio; CI, confidence interval.

Meta-analysis of the two studies (12, 17) involving 1878 participants (**Figure 3**) indicated that vitamin D supplementation does not seem to significantly affect the risk of developing T1DM (RR=0.66, 95%CI: 0.41-1.06) despite individual studies suggesting benefits. The 95% confidence interval for the individual studies are borderline significant independently but insignificant when pooled. This discrepancy could be due to the heterogeneity of both studies. Tenconi et al. focused on vitamin D supplementation during lactation while Stene et al. focused on vitamin D supplementation in the first year of life (2, 17). Both studies were also based in different countries and the small population size likely resulted in limited statistical power to detect a significant effect.

**Figure 4:**
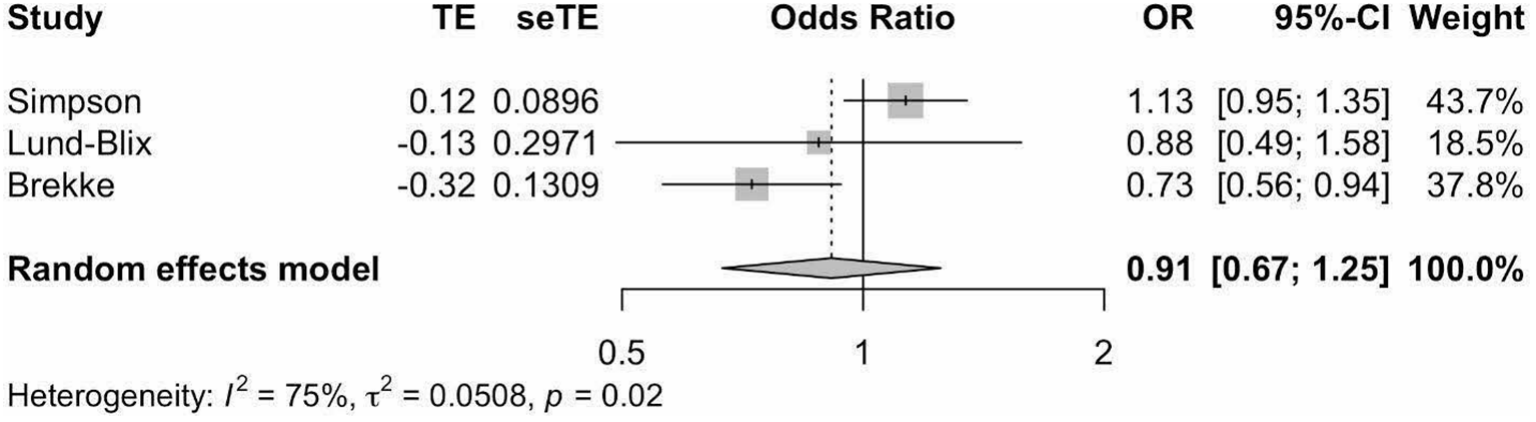
Precalculated pooled odds ratio of vitamin D supplementation on islet autoimmunity. TE, estimate of treatment effect; seTE, standard error of treatment estimate; OR, odds ratio; CI, confidence interval.

Meta-analysis of the three studies (18, 20, 25) (**Figure 4**) indicated that vitamin D supplementation does not seem to significantly affect the odds of developing IA (Pooled OR=0.91, 95%CI: 0.67-1.25).

### Progression of IA to T1DM

Three studies investigated the relationship between supplementation of different vitamin types and risk of progression of IA to T1DM (**Supplementary Table 2**) (11, 18, 28). Hakola et al. did not observe any associations between supplementation of any of the B vitamins and risk of progression from IA to T1DM in a cohort study with 344 children with IA (11). In a cohort study of 175 children with IA, Elhassan et al. found that multivitamin supplementation did not significantly influence the impact of lowering the risk of IA progression to T1DM, despite high iron intake (28). Simpson et al. reported that vitamin D supplementation did not significantly affect the risk of progression from IA to T1DM in 178 children (18).

### Systematic review

We systematically reviewed the confounding effects of vitamin D supplementation dosage and blood concentration on the relationship between vitamin D supplementation on the development of IA, T1DM or progression of IA to T1DM. This relationship was also explored in other vitamin types.

#### Vitamin D supplementation dosage

Two studies investigated the link between specific vitamin D dosages and the risk of developing T1DM or IA (**Supplementary Table 3**) (8, 19). Hyponnen et al. reported that the recommended vitamin D dosage of 2000IU, whether higher or lower than the recommended amount, did not significantly affect the risk of developing T1DM (8). Marjamaki et al. showed that the risk of developing T1DM or IA does not significantly differ between the 1^st^ and 4th quartile of vitamin D intake (19).

#### Vitamin D blood concentration

Two studies explored the association between vitamin D blood concentration and the risk of developing T1DM or IA (**Supplementary Table 4**) (18, 22). Gorham et al. found that the risk of having insulin-required diabetes was 3.5 times higher in individuals with the lowest 25(OH)D concentration compared to those with the highest concentration (22). On the contrary, Simpson et al. found that neither 25(OH)D nor vitamin D concentration was associated with the risk of developing IA or risk of progression of IA to T1DM (18).

#### Vitamin B

Two studies investigated the association between Vitamin B supplementation and the risk of T1DM, IA or progression of IA to T1DM (**Supplementary Table 5**) (11, 23). Hakola et al. reported that thiamine, riboflavin, niacin, pantothenic acid, pyridoxine, vitamin B12 supplementation were not significantly associated with the risk of developing IA or progression of IA to T1DM (11). Similarly, Gale et al. found that nicotinamide use was not significantly correlated with the risk of developing T1DM (23).

### Confounding factors affecting maternal supplementation

Our study systematically reviewed the prognostic factors affecting maternal usage of vitamin supplementation. These factors include demographic variables such as maternal characteristics, gender and age.

#### Maternal education level

Three studies explored the relationship between maternal education level and supplementation (**Supplementary Table 6**) (19, 25, 31). Hyponnen et al. reported that having no or basic maternal education was significantly associated with lower amounts of vitamin D supplementations in offsprings (31). Similarly, Marjamaki and Brekke et al. demonstrated a positive association between having a higher academic status with increased intake of vitamin D from food and supplements (19, 25).

#### Maternal age

Three studies investigated the link between maternal age and supplementation (**Supplementary Table 6**) (19, 25, 31). Brekke and Marjamaki et al. reported a significant association between higher maternal age and vitamin D supplementation (19, 25). Conversely, Hyponnen et al. revealed that older mothers were more likely to irregularly supplement their children’s diet with vitamin D (31).

#### Maternal Parity

Two studies explored the association between parity and supplementation (**Supplementary Table 6**) (19, 31). Hyponnen et al. reported that multiparity had a significant association with irregular supplementation of vitamin D in offspring (31). Conversely, Marjamaki et al. showed that primiparity had a significantly positive association with vitamin D supplementation (19).

#### Gender

Five studies explored the association between gender and supplementation (**Supplementary Table 7**) (11, 12, 17, 23, 24). Brikhou et al. reported a significantly association between being male and vitamin D intake (24). Both Hakola and Tenconi et al. showed that being a female was a predictive factor for pyridoxine intake and the risk of islet autoimmunity and T1DM (11, 17) Gale and Stene et al. found that sex made no significant difference on the association between nicotinamide treatment supplementation and the risk of developing T1DM (12, 23).

#### Participant Age

Three studies investigated the link between age and supplementation (**Supplementary Table 8**) (17, 23, 24). Brikhou et al. reported that age did not significantly affect vitamin D supplementation in both T1DM patients and healthy controls (24). Gale et al. revealed similar findings with nicotinamide treatment and the risk of developing T1DM (23). Conversely, Tenconi et al. showed that younger age was associated with higher levels of vitamin D supplementation and a lower risk of T1DM (17).

#### Risk-of-bias and publication bias

The quality of the methodology of the 15 studies was scored using the JBI checklist (**Supplementary Table 9**). No risk of bias was identified. Sensitivity analyses using leave-one-out and outlier analyses showed no singular studies that would affect the overall results (**Supplementary Figures 1–4**).

## Discussion

This systematic review and meta-analysis aimed to investigate the modifiable risk of developing IA, T1DM, progression of IA to T1DM with supplementation of vitamins. Overall, our results demonstrated that supplementation of vitamin D and B may not have any modulatory effect on disease risk but demographic factors such as a higher maternal education level increased levels of supplementation among mothers. Meta-analyses demonstrated that vitamin D supplementation might not have any effect on modifying the risk of developing IA or T1DM. Systematic review revealed that vitamin B supplementation did not influence the risk of T1DM and progression of IA to T1DM. Additionally, there was an association between higher maternal education levels and higher levels of vitamin D supplementation in their offspring. Despite this being a systematic review and meta-analysis of the available literature, there is considerable heterogeneity among the studies, complicating the ability to draw a definitive conclusion regarding the efficacy of vitamin supplementation in modifying the risk of developing IA, T1DM or progression of IA to T1DM.

It is important to note that the studies included in our analysis demonstrated considerable heterogeneity in their design, methodology and results. For instance, while the EURODIAB study demonstrated a protective role of vitamin D supplementation in reducing T1DM risk (27), other studies failed to replicate these findings. These discrepancies could be attributed to several factors. This includes differences in demographics in study populations, as the studies are carried out in different countries. The dosage and duration of supplementation, timing of intervention, and methods of measuring vitamin D status also varied across different studies. Furthermore, the definition and assessment of outcomes (IA, T1DM) was defined differently across studies, potentially contributing to the inconsistent results.

In recent years, emerging research has suggested that vitamin D supplementation in early childhood could play a pivotal role in modifying the risk of developing T1DM later in life, due to its potential anti-inflammatory and immunomodulatory effects (32). The EURODIAB study demonstrated the potential protective role of vitamin D supplementation in significantly reducing the risk of developing T1DM (27). Significantly, a study by Janner et al. revealed a high prevalence of vitamin D deficiency in children or adolescents with T1DM, suggesting an association between vitamin D levels and the risk of developing T1DM (33). Despite the prior hypotheses from existing literature, our results showed that vitamin D supplementation does not significantly affect the odds and RR of developing T1DM. While vitamin D might play a role in the immune system (34), it is important to recognize that T1DM is a multifactorial disease with a complicated pathogenesis (35). Other genetic, epigenetic, environmental and immunological factors contribute to disease development and progression (36), and might undermine the protective abilities of vitamin D. Another possibility is that only specific subgroups, for example those with severe vitamin D deficiency, may benefit from supplementation. As our included studies mainly looked at broad populations, this might not have been adequately captured in the analyses. As mentioned above, the heterogeneity of the studies must be considered as well. Firstly, the timing and duration of supplementation varied across studies, with some focusing on early infancy while others extended into childhood. Secondly, the dosage of vitamin D supplementation differed, ranging from 400 IU to over 2000IU daily. Additionally, the baseline vitamin D levels of participants varied across studies, which might have affected the efficacy of supplementation. These variations in intervention protocols could significantly impact the efficacy of supplementation Our results underscore the need for more targeted research, focusing on high-risk groups and considering other confounding factors to fully elucidate the relationship between vitamin D supplementation and T1DM risk.

Other than T1DM, our results further indicate that vitamin D supplementation does not seem to significantly affect the odds of developing IA or its progression to T1DM. Prior research has drawn possible links between vitamin D supplementation and a decreased risk of developing IA later in life, such as the study by Norris et al. (37 *)*, which determined that increased childhood plasma 25-hydroxyvitamin D concentration was associated with a decreasing IA risk. Similar to T1DM, it is imperative to realize that IA is also a multifactorial disease that can be influenced by environment, genetics, epigenetics, disease phenotypes and many other factors (38). The lack of a significant effect in our meta-analysis could be attributed to this, or variations in the physiology of the participants of each study. The heterogeneity of the studies included in the meta-analysis should also be kept in consideration, with variations in study design and population characteristics leading to limited ability to detect sub-group specific effects.

The role of vitamin B supplementation in modulating the risk of developing IA, T1DM or progression of IA to T1DM was also considered in our analysis. Our results concluded that vitamin B supplementation does not seem to significantly affect the odds. While the role of vitamin B in modulating immune responses is less well researched than vitamin D, vitamin B is postulated to have anti-inflammatory effects (39). The Boston Puerto Rican Health Study demonstrated that low vitamin B6 concentrations are associated with inflammation, higher oxidative stress, and metabolic conditions in older adults (40). Out of all the studies included in our meta-analysis, only two reported on vitamin B supplementation and the risk of developing IA or T1DM. The lack of significant findings could be attributed to the insufficient data and the variability in study designs, which may have obscured potential effects. While the current evidence from our study does not support a significant role for vitamin B in preventing T1DM or IA, further research with larger sample sizes and more robust study designs is necessary to explore this potential relationship more comprehensively.

Our results also revealed an association between demographic factors and disease risk, namely maternal education levels. Higher maternal education levels were correlated with higher supplementation of vitamin D in their offspring. This finding demonstrates how socio-economic and educational disparities can directly influence health literacy (41). Significantly, a study by Viinikainen et al. affirmed that individuals with higher educational levels were not only less prone to risky health behaviors such as smoking, but also allocated more attention to healthy habits, for instance the consumption of fruit and vegetables (42). In the context of this study, more educated mothers potentially had greater access to health information and resources, leading to higher rates of supplementation. This finding emphasizes on the importance of public health initiatives to tackle educational and socio-economic barriers to increase health literacy in the general population, particularly in underprivileged communities. This finding emphasizes that maternal education levels, which influence vitamin D supplementation in offspring, may play a critical role in shaping early-life health outcomes that affect the risk of developing T1DM. Several studies have demonstrated an association between lower maternal education level and higher risk of developing T1DM (43, 44). Andersson et al. found that in a cohort of 16,365 Swedish parents, lower maternal education level was associated with an increased risk of developing T1DM (43). Similarly, in a cohort of 4647 Norwegian children, Ruiz et al. demonstrated that the children of mothers with a master’s degree had a lower risk of type 1 diabetes mellitus than children of mothers with only a completed upper secondary education (44). Adequate vitamin D levels during critical developmental periods could contribute to immune system modulation, potentially reducing the likelihood of autoimmune conditions like T1DM, particularly in populations with higher maternal health literacy. Additionally, stratification for socio-economic factors that could directly affect health outcomes should also be highlighted in future studies.

The inconsistencies in our findings compared to some previous studies highlight the complex nature of vitamin D’s role in T1DM and IA. Future research should aim to address these discrepancies by conducting large-scale, long-term randomized controlled trials with standardized protocols for vitamin supplementation and outcome assessment. Additionally, studies should consider stratifying participants based on baseline vitamin D status, genetic risk factors, and other relevant variables to identify subgroups that may benefit most from supplementation. A majority of studies included in our review examined vitamin D. Further studies of other vitamins which have an immunomodulatory or antioxidative effect would be beneficial to elucidate the role of micronutrients in the pathophysiology of T1DM and IA (45–49).

### Limitations

This systematic review and meta-analysis should be considered in view of its limitations. Firstly, the heterogeneity of the included studies such as demographics factors, method of supplementation and study design could affect the outcomes observed. Differences in timing of supplementation, dosage (50), vitamin blood concentration, baseline dietary status and other factors could have affected the results. We recommend future studies to include data on important confounding factors such as vitamin levels and dosage information to gain a more comprehensive understanding of the effect of supplementation on disease risk. Different supplementation regimes for high-risk groups can also be explored. Secondly, due to insufficient data, we were unable to perform further subgroup meta-analyses and have adopted the synthesis without a meta-analyses approach. The small sample size across most studies could have potentially obscured nuanced effects in specific populations, particularly vulnerable ones (e.g., those with a baseline vitamin D deficiency). Future large-scale multi-national studies should be performed to investigate these effects in greater detail. Lastly, a few papers have investigated the link of the vitamin D receptor gene and T1DM (51, 52), but there has been no conclusive evidence of its direct effect, hence we were unable to draw any meaningful conclusions regarding this association.

## Conclusion

Our study demonstrated that the available literature presents inconsistent findings, likely due to substantial heterogeneity which complicates the ability to draw definitive conclusions regarding the efficacy of vitamin B and D supplementation in modifying the risk of developing IA, T1DM or progression of IA to T1DM. The data suggests that demographic factors can have an impact on T1DM risk. Vitamin D deficiencies are linked to a higher risk of developing T1DM. Vitamin D deficiency may be less likely in children to mothers with higher educational levels and health literacy, due to higher rates of vitamin D supplementation. This finding has important implications for public health initiatives, particularly when considering implementing prophylactic supplement programs. Our study provides a foundation for future research by contributing to the evolving landscape of nutritional immunology.

## Data Availability

The original contributions presented in the study are included in the article/**Supplementary Material**. Further inquiries can be directed to the corresponding authors.

## References

[1] Singhealth. Available online at: https://www.singhealth.com.sg/patient-care/conditions-treatments/Type-1-diabetes-mellitus (Accessed February 1, 2025).

[2] Stene LC Barriga K Norris JM Hoffman M Erlich HA Eisenbarth GS . Perinatal factors and development of islet autoimmunity in early childhood: the diabetes autoimmunity study in the young. Am J Epidemiol. (2004) 160:3–10. doi: 10.1093/aje/kwh159 15229111

[3] Ogrotis I Koufakis T and Kotsa K . Changes in the global epidemiology of type 1 diabetes in an evolving landscape of environmental factors: causes, challenges, and opportunities. Medicina (Kaunas). (2023) 59. doi: 10.3390/medicina59040668 PMC10141720 37109626

[4] White NH . Long-term outcomes in youths with diabetes mellitus. Pediatr Clin North Am. (2015) 62:889–909. doi: 10.1016/j.pcl.2015.04.004 26210623 PMC4662600

[5] Nielsen HB Ovesen LL Mortensen LH Lau CJ Joensen LE . Type 1 diabetes, quality of life, occupational status and education level &x2013; A comparative population-based study. Diabetes Res Clin Pract. (2016) 121:62–8. doi: 10.1016/j.diabres.2016.08.021 27662040

[6] Sharif K Watad A Coplan L Amital H Shoenfeld Y Afek A . Psychological stress and type 1 diabetes mellitus: what is the link? Expert Rev Clin Immunol. (2018) 14:1081–8. doi: 10.1080/1744666x.2018.1538787 30336709

[7] Joish VN Zhou FL Preblick R Lin D Deshpande M Verma S . Estimation of annual health care costs for adults with type 1 diabetes in the United States. J Manag Care Spec Pharm. (2020) 26:311–8. doi: 10.18553/jmcp.2020.26.3.311 PMC10390990 32105172

[8] Hyppönen E Läärä E Reunanen A Järvelin M-R Virtanen SM . Intake of vitamin D and risk of type 1 diabetes: a birth-cohort study. Lancet. (2001) 358:1500–3. doi: 10.1016/S0140-6736(01)06580-1 11705562

[9] Carakushansky M Patel P Ben Khallouq BA Gurnurkar S . Prevalence of vitamin D deficiency in children with type 1 diabetes mellitus. Cureus. (2020) 12:e7836. doi: 10.7759/cureus.7836 32467811 PMC7250523

[10] Saggese G Federico G Balestri M Toniolo A . Calcitriol inhibits the PHA-induced production of IL-2 and IFN-gamma and the proliferation of human peripheral blood leukocytes while enhancing the surface expression of HLA class II molecules. J Endocrinol Invest. (1989) 12:329–35. doi: 10.1007/bf03349999 2504804

[11] Hakola L Mramba LK Uusitalo U Andrén Aronsson C Hummel S Niinistö S . Intake of B vitamins and the risk of developing islet autoimmunity and type 1 diabetes in the TEDDY study. Eur J Nutr. (2024) 63:1329–38. doi: 10.1007/s00394-024-03346-6 PMC11139689 38413484

[12] Stene LC Joner G . Use of cod liver oil during the first year of life is associated with lower risk of childhood-onset type 1 diabetes: a large, population-based, case-control study. Am J Clin Nutr. (2003) 78:1128–34. doi: 10.1093/ajcn/78.6.1128 14668274

[13] Johnson RK Vanderlinden LA DeFelice BC Uusitalo U Seifert J Fan S . Metabolomics-related nutrient patterns at seroconversion and risk of progression to type 1 diabetes. Pediatr Diabetes. (2020) 21:1202–9. doi: 10.1111/pedi.13085 PMC7855902 32686271

[14] Julian Higgins JT . Cochrane handbook for systematic reviews of interventions. Chichester, UK: John Wiley & Sons (2022).

[15] Higgins JP Thompson SG . Quantifying heterogeneity in a meta-analysis. Stat Med. (2002) 21:1539–58. doi: 10.1002/sim.1186 12111919

[16] Munn Z Barker TH Moola S Tufanaru C Stern C McArthur A . Methodological quality of case series studies: an introduction to the JBI critical appraisal tool. JBI Evidence Synthesis. (2020) 18. doi: 10.11124/JBISRIR-D-19-00099 33038125

[17] Tenconi MT Devoti G Comelli M Pinon M Capocchiano A Calcaterra V . Major childhood infectious diseases and other determinants associated with type 1 diabetes: a case-control study. Acta Diabetol. (2007) 44:14–9. doi: 10.1007/s00592-007-0235-9 17357880

[18] Simpson M Brady H Yin X Seifert J Barriga K Hoffman M . No association of vitamin D intake or 25-hydroxyvitamin D levels in childhood with risk of islet autoimmunity and type 1 diabetes: the Diabetes Autoimmunity Study in the Young (DAISY). Diabetologia. (2011) 54:2779–88. doi: 10.1007/s00125-011-2278-2 PMC3478880 21858504

[19] Marjamäki L Niinistö S Kenward MG Uusitalo L Uusitalo U Ovaskainen ML . Maternal intake of vitamin D during pregnancy and risk of advanced beta cell autoimmunity and type 1 diabetes in offspring. Diabetologia. (2010) 53:1599–607. doi: 10.1007/s00125-010-1734-8 20369220

[20] Lund-Blix NA Stene LC Rasmussen T Torjesen PA Andersen LF Rønningen KS . Infant feeding in relation to islet autoimmunity and type 1 diabetes in genetically susceptible children: the MIDIA Study. Diabetes Care. (2015) 38:257–63. doi: 10.2337/dc14-1130 25422170

[21] Jacobsen R Hypponen E Sørensen TI Vaag AA Heitmann BL . Gestational and early infancy exposure to margarine fortified with vitamin D through a national danish programme and the risk of type 1 diabetes: the D-tect study. PloS One. (2015) 10:e0128631. doi: 10.1371/journal.pone.0128631 26030061 PMC4452099

[22] Gorham ED Garland CF Burgi AA Mohr SB Zeng K Hofflich H . Lower prediagnostic serum 25-hydroxyvitamin D concentration is associated with higher risk of insulin-requiring diabetes: a nested case-control study. Diabetologia. (2012) 55:3224–7. doi: 10.1007/s00125-012-2709-8 22955995

[23] Gale EA Bingley PJ Emmett CL Collier T . European Nicotinamide Diabetes Intervention Trial (ENDIT): a randomised controlled trial of intervention before the onset of type 1 diabetes. Lancet. (2004) 363:925–31. doi: 10.1016/s0140-6736(04)15786-3 15043959

[24] Brikhou S Nouari W Bouazza S Mezouar CE Benzian Z Talha K . Dietary vitamin D intake and sun exposure are not associated with type 1 diabetic schoolchildren and adolescents: a first report in Algerian Sahara. medRxiv. (2022). doi: 10.1101/2022.07.17.22276883

[25] Brekke HK Ludvigsson J . Vitamin D supplementation and diabetes-related autoimmunity in the ABIS study. Pediatr Diabetes. (2007) 8:11–4. doi: 10.1111/j.1399-5448.2006.00223.x 17341286

[26] Awadalla NJ Hegazy AA Abd-El-Salam M Elhady M . Environmental factors associated with type 1 diabetes development: A case control study in Egypt. Int J Environ Res Public Health. (2017) 14. doi: 10.3390/ijerph14060615 PMC5486301 28590451

[27] Vitamin D supplement in early childhood and risk for Type I (insulin-dependent) diabetes mellitus. The EURODIAB Substudy 2 Study Group. Diabetologia. (1999) 42:51–4. doi: 10.1007/s001250051112 10027578

[28] Elhassan S Dong F Buckner T Johnson RK Seifert JA Carry PM . Investigating iron intake in risk of progression from islet autoimmunity to type 1 diabetes: The diabetes autoimmunity study in the young. Front Immunol. (2023) 14. doi: 10.3389/fimmu.2023.1124370 PMC10086157 37056761

[29] Koivusaari K Syrjälä E Niinistö S Takkinen H-M Ahonen S Åkerlund M . Consumption of differently processed milk products in infancy and early childhood and the risk of islet autoimmunity. Br J Nutr. (2020) 124:173–80. doi: 10.1017/S0007114520000744 32102698

[30] Thorsen SU Halldorsson TI Bjerregaard AA Olsen SF Svensson J . Maternal and early life iron intake and risk of childhood type 1 diabetes: A danish case-cohort study. Nutrients. (2019). doi: 10.3390/nu11040734 PMC6521102 30934897

[31] Hyppönen E Läärä E Reunanen A Järvelin MR Virtanen SM . Intake of vitamin D and risk of type 1 diabetes: a birth-cohort study. Lancet. (2001) 358:1500–3. doi: 10.1016/s0140-6736(01)06580-1 11705562

[32] Wimalawansa SJ . Infections and autoimmunity-the immune system and vitamin D: A systematic review. Nutrients. (2023). doi: 10.3390/nu15173842 PMC10490553 37686873

[33] Janner M Ballinari P Mullis PE Flück CE . High prevalence of vitamin D deficiency in children and adolescents with type 1 diabetes. Swiss Med Wkly. (2010) 140:w13091. doi: 10.4414/smw.2010.13091 20853194

[34] Prietl B Treiber G Pieber TR Amrein K . Vitamin D and immune function. Nutrients. (2013) 5:2502–21. doi: 10.3390/nu5072502 PMC3738984 23857223

[35] Dotta F Fondelli C and Di Mario U . Type 1 diabetes mellitus as a polygenic multifactorial disease: immunopathogenic mechanisms of beta-cell destruction. Acta BioMed. (2005) 76 Suppl 3:14–8. 16915790

[36] Zajec A Trebušak Podkrajšek K Tesovnik T Šket R Čugalj Kern B Jenko Bizjan B . Pathogenesis of type 1 diabetes: established facts and new insights. Genes. (2022) 13. doi: 10.3390/genes13040706 PMC9032728 35456512

[37] Norris JM Lee H-S Frederiksen B Erlund I Uusitalo U Yang J . Plasma 25-hydroxyvitamin D concentration and risk of islet autoimmunity. Diabetes. (2017) 67:146–54. doi: 10.2337/db17-0802 PMC5741144 29061729

[38] Ziegler A-G Meier-Stiegen F Winkler C Bonifacio E the TSG . Prospective evaluation of risk factors for the development of islet autoimmunity and type 1 diabetes during puberty – TEENDIAB: study design. Pediatr Diabetes. (2012) 13:419–24. doi: 10.1111/j.1399-5448.2011.00763.x 21446926

[39] Mikkelsen K Dargahi N Fraser S Apostolopoulos V . High-dose vitamin B6 (Pyridoxine) displays strong anti-inflammatory properties in lipopolysaccharide-stimulated monocytes. Biomedicines. (2023) 11. doi: 10.3390/biomedicines11092578 PMC10526783 37761018

[40] Shen J Lai C-Q Mattei J Ordovas JM Tucker KL . Association of vitamin B-6 status with inflammation, oxidative stress, and chronic inflammatory conditions: the Boston Puerto Rican Health Study123. Am J Clin Nutr. (2010) 91:337–42. doi: 10.3945/ajcn.2009.28571 PMC2806890 19955400

[41] Yuen E Winter N Savira F Huggins CE Nguyen L Cooper P . Digital health literacy and its association with sociodemographic characteristics, health resource use, and health outcomes: rapid review. Interact J Med Res. (2024) 13:e46888. doi: 10.2196/46888 39059006 PMC11316163

[42] Viinikainen J Bryson A Böckerman P Kari JT Lehtimäki T Raitakari O . Does better education mitigate risky health behavior? A mendelian randomization study. Econ Hum Biol. (2022) 46:101134. doi: 10.1016/j.ehb.2022.101134 35354116

[43] White PA Faresjö T Jones MP Ludvigsson J . Low maternal education increases the risk of Type 1 Diabetes, but not other autoimmune diseases: a mediating role of childhood BMI and exposure to serious life events. Sci Rep. (2023) 13:6166. doi: 10.1038/s41598-023-32869-x 37061552 PMC10105777

[44] Lopez-Doriga-Ruiz P Tapia G Bakken IJ Håberg SE Gulseth HL Skrivarhaug T . Parental education and occupation in relation to childhood type 1 diabetes: nationwide cohort study. J Epidemiol Community Health. (2024) 78:319–25. doi: 10.1136/jech-2023-220693 38302277

[45] Mattila M Erlund I Lee H-S Niinistö S Uusitalo U Andrén Aronsson C . Plasma ascorbic acid and the risk of islet autoimmunity and type 1 diabetes: the TEDDY study. Diabetologia. (2020) 63:278–86. doi: 10.1007/s00125-019-05028-z PMC6946743 31728565

[46] Gupta S Sharma TK Kaushik GG Shekhawat VP . Vitamin E supplementation may ameliorate oxidative stress in type 1 diabetes mellitus patients. Clin Lab. (2011) 57:379–86. 21755829

[47] Lee ARYB Tariq A Lau G Tok NWK Tam WWS Ho CSH . Vitamin E alpha-tocopherol, and its effects on depression and anxiety: a systematic review and meta-analysis. Nutrients. (2022) 14:656. doi: 10.3390/nu14030656 35277015 PMC8840247

[48] Trasino SE Gudas LJ . Vitamin A: a missing link in diabetes? Diabetes Manag (Lond). (2015) 5:359–67. doi: 10.2217/dmt.15.30 PMC4623591 26535059

[49] Yosaee S Akbari Fakhrabadi M Shidfar F . Positive evidence for vitamin A role in prevention of type 1 diabetes. World J Diabetes. (2016) 7:177–88. doi: 10.4239/wjd.v7.i9.177 PMC4856890 27162582

[50] Low CE Loke S Chew NSM Lee ARYB Tay SH . Vitamin, antioxidant and micronutrient supplementation and the risk of developing incident autoimmune diseases: a systematic review and meta-analysis. Front Immunol. (2024) 15:1453703. doi: 10.3389/fimmu.2024.1453703 39717776 PMC11663920

[51] Mostafa EA Abo Hashish MMA Ismail NA Hasanin HM Hasanin RM Wahby AA . Assessment of vitamin D status and vitamin D receptor polymorphism in Egyptian children with Type 1 diabetes. J Genet Eng Biotechnol. (2024) 22:100343. doi: 10.1016/j.jgeb.2023.100343 38494252 PMC10980865

[52] Zhai N Bidares R Makoui MH Aslani S Mohammadi P Razi B . Vitamin D receptor gene polymorphisms and the risk of the type 1 diabetes: a meta-regression and updated meta-analysis. BMC Endocr Disord. (2020) 20:121. doi: 10.1186/s12902-020-00575-8 32771009 PMC7414991

