# Supplementary material for: Vitamin supplementation and its effect on incident type 1 diabetes mellitus and islet autoimmunity: a systematic review and meta-analysis
Source: Front Immunol. 2025 Apr 9;16:1505324. doi: 10.3389/fimmu.2025.1505324 (PMC12014702; doi:10.3389/fimmu.2025.1505324)

**Vitamin Supplementation and its Effect on Incident Type 1 Diabetes Mellitus and Islet Autoimmunity: A Systematic Review and Meta-analysis**

Supplementary Table 1: Search strategy

Supplementary Table 2: Evaluation of the mediating effects of supplementation among different vitamins on the risk of Type 1 Diabetes Mellitus, Islet Autoimmunity or progression of Islet Autoimmunity to Type 1 Diabetes Mellitus

Supplementary Table 3: Evaluation of the association between specific vitamin D dosages and the risk of developing Type 1 Diabetes Mellitus or Islet Autoimmunity

Supplementary Table 4: Evaluation of the association between serum vitamin D concentration and the risk of developing Type 1 Diabetes Mellitus or Islet Autoimmunity

Supplementary Table 5: Evaluation of the mediating or confounding effect of the types of vitamin B on the risk of Type 1 Diabetes Mellitus, Islet Autoimmunity or progression of Islet Autoimmunity to Type 1 Diabetes Mellitus

Supplementary Table 6: Evaluation of the mediating or confounding effect of maternal factors on vitamins and the risk of Type 1 Diabetes Mellitus, Islet Autoimmunity or progression of Islet Autoimmunity to Type 1 Diabetes Mellitus

Supplementary Table 7: Evaluation of the mediating or confounding effect of gender on vitamins and the risk of Type 1 Diabetes Mellitus, Islet Autoimmunity or progression of Islet Autoimmunity to Type 1 Diabetes Mellitus

Supplementary Table 8: Evaluation of the mediating or confounding effect of age on vitamins and the risk of Type 1 Diabetes Mellitus, Islet Autoimmunity or progression of Islet Autoimmunity to Type 1 Diabetes Mellitus

Supplementary Table 9: Quality assessment of included cohort studies using the Joanna Brigg’s Institute Critical Appraisal tool

Supplementary Figure 1: Leave-one-out analysis of studies assessing the pooled odds ratio of vitamin D supplementation and Type 1 Diabetes Mellitus

Supplementary Figure 2: Outlier assessment of studies assessing the pooled odds ratio of vitamin D supplementation and Type 1 Diabetes Mellitus

Supplementary Figure 3: Leave-one-out analysis of studies assessing the pooled odds ratio of vitamin D supplementation and Islet Autoimmunity

Supplementary Figure 4: Outlier assessment of studies assessing the pooled odds ratio of vitamin D supplementation and Islet Autoimmunity

Supplementary Table 1: Search strategy

**Embase**

| #1 | ('vitamin supplementation'/exp OR 'vitamin intake'/exp OR 'micronutrient intake'/exp OR 'multivitamin'/exp OR 'vitamin*':ti,ab OR 'antioxidant*':ti,ab) |
| --- | --- |
| #2 | 'insulin dependent diabetes mellitus'/exp OR 'autoimmune diabetes'/exp OR 'juvenile diabetes':ti,ab OR 'T1DM':ti,ab OR ‘Type 1 Diabetes Mellitus’:ti,ab OR ‘Islet Autoimmunity’ |
| #3 | ('incidence*':ti,ab OR 'risk*':ti,ab OR 'prevalence*':ti,ab) |

#1 and #2 and #3
Limited to Year 2000

**PubMed**

| #1 | ("Micronutrients"[Mesh] OR "Vitamins"[Mesh] OR "Antioxidants"[Mesh] OR "Micronutrient*"[Title/Abstract] OR “Vitamin*”[Title/Abstract] OR "Antioxida*"[Title/Abstract]) |
| --- | --- |
| #2 | "Diabetes Mellitus, Type 1"[Mesh] OR “Diabetes Mellitus, Insulin-Dependent”[Title/Abstract] OR “Diabetes Mellitus, Insulin Dependent”[Title/Abstract] OR “Insulin-Dependent Diabetes Mellitus”[Title/Abstract] OR “Juvenile-Onset Diabetes Mellitus”[Title/Abstract] OR “Juvenile Onset Diabetes”[Title/Abstract] OR “IDDM”[Title/Abstract] OR “Type 1 Diabetes*”[Title/Abstract] OR “Autoimmune Diabetes”[Title/Abstract] OR “T1DM”[Title/Abstract] OR ‘Islet Autoimmunity’ |
| #3 | ('incidence*'[Title/Abstract] OR 'risk*[Title/Abstract] OR 'prevalence*'[Title/Abstract]) |

#1 and #2 and #3
Limited to Year 2000

Supplementary Table 2: Evaluation of the mediating effects of supplementation among different vitamins on the risk of Type 1 Diabetes Mellitus, Islet Autoimmunity or progression of Islet Autoimmunity to Type 1 Diabetes Mellitus

| **Author** | **Year** | **Country** | **Study population** | **Key findings†** |
| --- | --- | --- | --- | --- |
| Hakola | 2024 | US, Finland, Sweden, Germany | 344 T1DM-susceptible children with IA born in the U.S., Finland, Sweden, and Germany in 2004-2010 were recruited as part of the Environmental Determinants of Diabetes in the Young (TEDDY) study. | No associations between supplementation of any of the B vitamins and risk of progression from IA to T1DM.  Thiamin (HR=0.95, 95%CI: 0.62-1.43), Riboflavin (HR=0.91, 95%CI: 0.64-1.29), Niacin (HR=1.00, 95%CI: 0.96-1.03), Pantothenic (HR=0.99, 95%CI: 0.87-1.11), Pyridoxine (HR=1.14, 95%CI: 0.84-1.54), Vitamin B12 (HR=0.95, 95%CI: 0.85-1.05). |
| Elhassan | 2023 | USA | 175 children with IA were evaluated for risk of progression of IA to T1DM, as part of the DAISY cohort study. | No associations between multivitamin supplementation and risk of progression from IA to T1DM (HR=0.80, 95%CI: 0.49-1.32). |
| Simpson | 2011 | USA | 198 children with IA were evaluated for risk of progression of IA to T1DM, as part of the DAISY cohort study. | No associations between vitamin D supplementation and risk of progression from IA to T1DM (HR=1.30, 95%CI: 0.91-1.86). |

Abbreviations: SD, standard deviation; OR, odds ratio; RR, risk ratio; CI, confidence interval

†Outcomes of interest include logistic or linear regression analysis for any association between vitamins on the risk of Type 1 Diabetes Mellitus, Islet Autoimmunity or progression of Islet Autoimmunity to Type 1 Diabetes Mellitus

Supplementary Table 3: Evaluation of the association between specific vitamin D dosages and the risk of developing Type 1 Diabetes Mellitus or Islet Autoimmunity

| **Author** | **Year** | **Country** | **Study population** | **Key findings†** |
| --- | --- | --- | --- | --- |
| Hyponnen | 2021 | Finland | 12055 pregnant women were recruited as part of a birth-cohort study in Finland. | Children who regularly took the recommended dose of vitamin D (2000 IU daily) had a RR of 0.22, 95%CI: 0.05-0.89 compared with those who regularly received less than the recommended amount. |
| Marjamaki | 2010 | Finland | Mothers of 3723 infants born between  1997 and 2002 completed a validated 181-item food  frequency questionnaire in Finland. | The risk of developing T1DM or IA does not significantly differ between the 1^st^ quartile and the 4^th^ quartile of vitamin D intake (OR=1.15, 95%CI: 0.73–1.82). |

Abbreviations: SD, standard deviation; OR, odds ratio; RR, risk ratio; CI, confidence interval; T1DM, Type 1 Diabetes Mellitus; IA, Islet Autoimmunity

†Outcomes of interest include logistic or linear regression analysis for any association between age and the risk of Type 1 Diabetes Mellitus, Islet Autoimmunity or progression of Islet Autoimmunity to Type 1 Diabetes Mellitus

Supplementary Table 4: Evaluation of the association between serum vitamin D concentration and the risk of developing Type 1 Diabetes Mellitus or Islet Autoimmunity

| **Author** | **Year** | **Country** | **Study population** | **Key findings†** |
| --- | --- | --- | --- | --- |
| Simpson | 2011 | USA | 198 children with IA were evaluated for the risk of progression of IA to T1DM, as part of the DAISY cohort study. | No significant association between vitamin D concentration and risk of IA or T1DM (HR=1.16, 95%CI: 0.69-1.93). |
| Gorham | 2012 | USA | 1000 cases of insulin requiring diabetes amongst US military service members and an equal number of healthy controls were recruited as part of a case-control study in the USA. | The risk of having insulin-requiring diabetes was significantly higher in individuals with the lowest Vitamin D concentrations than the highest concentrations (OR=3.5, 95%CI: 2.0-6.0). |

Abbreviations: SD, standard deviation; OR, odds ratio; HR, hazard ratio; CI, confidence interval; T1DM, Type 1 Diabetes Mellitus; IA, Islet Autoimmunity

†Outcomes of interest include logistic or linear regression analysis for any association between age and the risk of Type 1 Diabetes Mellitus, Islet Autoimmunity or progression of Islet Autoimmunity to Type 1 Diabetes Mellitus

Supplementary Table 5: Evaluation of the mediating or confounding effect of the types of vitamin B on the risk of Type 1 Diabetes Mellitus, Islet Autoimmunity or progression of Islet Autoimmunity to Type 1 Diabetes Mellitus

| **Author** | **Year** | **Country** | **Study population** | **Dosage** | **Key findings†** |
| --- | --- | --- | --- | --- | --- |
| **Vitamin B** | | | | | |
| Hakola | 2024 | US, Finland, Sweden, Germany | 8500 T1DM-susceptible children born in the U.S., Finland, Sweden, and Germany in 2004-2010 were recruited as part of the Environmental Determinants of Diabetes in the Young (TEDDY) study. | mg/1000kcal (thiamine, riboflavin, niacin, pantothenic acid and pyridoxine). ug/1000kcal (Vitamin B12 and folate) | Thiamine (HR=0.95, 95%CI: 0.62-1.43), Riboflavin (OR=0.91, 95%CI: 0.64-1.29), Niacin (OR=1.00, 95%CI: 0.96-1.03), Pantothenic acid (OR=0.99, 95%CI: 0.87-1.11), Pyridoxine (OR=1.14, 95%CI: 0.84-1.54), Vitamin B12 (OR=0.95, 95%CI: 0.85-1.05) and Folate (OR=0.98, 95%CI: 0.94-1.01) were not significantly associated with the risk of progression of IA to T1DM.  Thiamine (HR=0.97, 95%CI: 0.72-1.31), Riboflavin (OR=1.01, 95%CI: 0.83-1.24), Niacin (OR=0.99, 95%CI: 0.96-1.01), Pantothenic acid (OR=1.00, 95%CI: 0.93-1.08), Pyridoxine (OR=0.81, 95%CI: 0.63-1.04), Vitamin B12 (OR=0.95, 95%CI: 0.89-1.02) and Folate (OR=1.00, 95%CI: 0.98-1.02) were not significantly associated with the risk of developing any IA.  Higher intake of niacin was associated with a decreased risk of developing multiple autoantibodies (OR=0.95, 95%CI: 0.92-0.98). Higher intake of pyridoxine (OR=0.66, 95%CI: 0.46-0.96) and vitamin B12 (OR=0.87, 95%CI: 0.77-0.97) was associated with a decreased risk of islet antigen autoantibody-first autoimmunity. Higher intake of riboflavin (OR=1.38, 95%CI: 1.05-1.80) associated with increased risk of glutamic acid decarboxylase autoantibody first autoimmunity  (Table 2). |
| Gale | 2004 | Multiple | 552 relatives with confirmed islet cell  antibody (ICA) levels of 20 Juvenile Diabetes Federation (JDF)  units or more were recruited as part of a double-blind placebo-controlled  Trial in multiple countries. | 1·2 g/m2 daily up to a maximum of  3 g/day | Nicotinamide use was not significantly correlated with the risk of developing T1DM (HR=1.01, 95%CI: 0.70-1.38). |

Abbreviations: CI, confidence interval; OR, odds ratio; RR, risk ratio; SD, standard deviation; NR, not reported

†Outcomes of interest include logistic or linear regression analysis for any association between different types of vitamins and the risk of Type 1 Diabetes Mellitus, Islet Autoimmunity or progression of Islet Autoimmunity to Type 1 Diabetes Mellitus

Supplementary Table 6: Evaluation of the mediating or confounding effect of maternal factors on vitamins and the risk of Type 1 Diabetes Mellitus, Islet Autoimmunity or progression of Islet Autoimmunity to Type 1 Diabetes Mellitus

| **Author** | **Year** | **Country** | **Study population** | **Key findings†** |
| --- | --- | --- | --- | --- |
| **Maternal education level** | | | | |
| Hyponnen | 2021 | Finland | 12055 pregnant women were recruited as part of a birth-cohort study in Finland. | Less educated mothers were more likely to irregularly supplement their children’s diets with vitamin D than well-educated mothers (p<0.001). |
| Marjamaki | 2010 | Finland | Mothers of 3723 infants born between  1997 and 2002 completed a validated 181-item food  frequency questionnaire in Finland. | Higher intake of vitamin D from food and supplements correlated positively with education of the mother (p<0.0001). |
| Brekke | 2007 | Sweden | 16070 infants were recruited as part of a cohort study in Norway. | Non supplementation of infants with AD drops at 2.5 years was not significantly associated with maternal education. A higher proportion of mothers who used vitamin-D-containing supplements had >12 years of education (48 vs. 31%, p<0.001). |
| **Maternal age** | | | | |
| Hyponnen | 2021 | Finland | 12055 pregnant women were recruited as part of a birth-cohort study in Finland. | Older mothers were more likely to irregularly supplement their children’s diets with vitamin D (p<0.05). |
| Marjamaki | 2010 | Finland | Mothers of 3723 infants born between  1997 and 2002 completed a validated 181-item food  frequency questionnaire in Finland. | Higher intake of vitamin D from food correlated positively with higher age of the mother (p <0.0001). |
| Brekke | 2007 | Sweden | 16070 infants were recruited as part of a cohort study in Norway. | Mothers who used vitamin-D-containing supplements during pregnancy were of a higher age (30 vs. 29 years, p<0.001). |
| **Maternal Parity** | | | | |
| Marjamaki et al. | 2010 | Finland | Mothers of 3723 infants born between  1997 and 2002 completed a validated 181-item food  frequency questionnaire in Finland. | Higher intake of vitamin D from supplements correlated positively with the mother being primiparous (p<0.05). |
| Hyponnen et al. | 2021 | Finland | 12055 pregnant women were recruited as part of a birth-cohort study in Finland. | Mothers with more children were more likely to irregularly supplement their children’s diets with vitamin D (p<0.05). |

Abbreviations: SD, standard deviation; OR, odds ratio; RR, risk ratio; CI, confidence interval

†Outcomes of interest include logistic or linear regression analysis for any association between maternal factors and risk of Type 1 Diabetes Mellitus, Islet Autoimmunity or progression of Islet Autoimmunity to Type 1 Diabetes Mellitus

Supplementary Table 7: Evaluation of the mediating or confounding effect of gender on vitamins and the risk of Type 1 Diabetes Mellitus, Islet Autoimmunity or progression of Islet Autoimmunity to Type 1 Diabetes Mellitus

| **Author** | **Year** | **Country** | **Study population** | **Key findings†** |
| --- | --- | --- | --- | --- |
| Brikhou | 2023 | Algeria | 335 type 1 individuals with diabetes and age- and gender-matched 41 healthy Algerian schoolchildren and teenagers were recruited as part of a case-control study in Algerian Sahara. | Being male was significantly correlated with higher vitamin D intake in individuals with diabetes as compared to non-individuals with diabetes (p<0.05). |
| Hakola | 2024 | US, Finland, Sweden, Germany | 8500 T1DM-susceptible children born in the U.S., Finland, Sweden, and Germany in 2004-2010 were recruited as part of the Environmental Determinants of Diabetes in the Young (TEDDY) study. | Pyridoxine intake tended to decrease the risk of GADA-first autoimmunity among girls (HR=0.56, 95%CI: 0.27-1.15) and increase it among boys (HR=1.34, 95%CI: 0.88-2.03), p for interaction=0.0496. |
| Stene | 2003 | Norway | 545 cases of childhood-onset type 1 diabetes and 1668  population control subjects were recruited as part of a case-control study in Norway. | The association of risk of diabetes with use of cod liver oil during the first year of life was somewhat stronger among girls than among boys, but was not statistically significant (p=0.06) |
| Gale | 2004 | Multiple | 552 relatives with confirmed islet cell antibody levels of 20 Juvenile Diabetes Federation (JDF) units or more were recruited as part of a double-blind placebo-controlled trial in multiple countries. | Sex made no significant difference on the association between nicotinamide treatment and the risk of developing diabetes (Males: p=0.97, Females p=0.53) |
| Tenconi | 2007 | Italy | 159 individuals with diabetes and 318 healthy controls were recruited for a case-control study in Italy. | Being female made a significant difference on the association between Vitamin D intake and T1DM risk (OR=0.14, 95%CI: 0.02-0.74). Being male made no significant difference on the association between Vitamin D intake and T1DM risk (OR=0.38, 95%CI: 0.12-1.21) |

Abbreviations: SD, standard deviation; OR, odds ratio; RR, risk ratio; CI, confidence interval

†Outcomes of interest include logistic or linear regression analysis for any association between gender and the risk of Type 1 Diabetes Mellitus, Islet Autoimmunity or progression of Islet Autoimmunity to Type 1 Diabetes Mellitus

Supplementary Table 8: Evaluation of the mediating or confounding effect of participant age on vitamins and risk of Type 1 Diabetes Mellitus or Islet Autoimmunity

| **Author** | **Year** | **Country** | **Study population** | **Key findings†** |
| --- | --- | --- | --- | --- |
| Brikhou | 2023 | Algeria | 335 type 1 individuals with diabetes and age- and gender-matched 41 healthy Algerian schoolchildren and teenagers were recruited as part of a case-control study in Algerian Sahara. | Age made no significant difference in vitamin D intake between T1DM patients and healthy controls (5-10 years old: p=0.268, 10-15 years old: p=0.388, >15 years old: p=0.185) |
| Gale | 2004 | Multiple | 552 relatives with confirmed islet cell  antibody (ICA) levels of 20 Juvenile Diabetes Federation (JDF) units or more were recruited as part of a double-blind placebo-controlled Trial in multiple countries. | Age made no significant difference on the association between nicotinamide treatment and the risk of developing diabetes (<20 years old: p=0.91, >20 years old: p=0.33) |
| Tenconi | 2007 | Italy | 159 individuals with diabetes and 318 healthy controls were recruited for a case-control study in Italy. | Being aged 0-14 as opposed to 15-29 years made a significant difference on the association between Vitamin D intake and T1DM risk (OR=0.31, 95%CI: 0.11-0.86) |

Abbreviations: SD, standard deviation; OR, odds ratio; RR, risk ratio; CI, confidence interval

†Outcomes of interest include logistic or linear regression analysis for any association between age and the risk of Type 1 Diabetes Mellitus, Islet Autoimmunity or progression of Islet Autoimmunity to Type 1 Diabetes Mellitus

Supplementary Table 9: Quality assessment of included cohort studies using the Joanna Brigg’s Institute Critical Appraisal tool

9.1: JBI Critical Appraisal Checklist for Cohort Studies

| Author | 1. Were the two groups similar and recruited from the same population? | 2. Were the exposures measured similarly to assign people to both exposed and unexposed groups? | 3. Was the exposure measured in a valid and reliable way? | 4. Were confounding factors identified? | 5. Were strategies to deal with confounding factors stated? | 6. Were the groups/participants free of the outcome at the start of the study (or at the moment of exposure)? | 7. Were the outcomes measured in a valid and reliable way? | 8. Was the follow up time reported and sufficient to be long enough for outcomes to occur? | 9. Was follow up complete, and if not, were the reasons to loss to follow up described and explored? | 10. Were strategies to address incomplete follow up utilized? | 11. Was appropriate statistical analysis used? | Total |
| --- | --- | --- | --- | --- | --- | --- | --- | --- | --- | --- | --- | --- |
| Jacobsen | Y | Y | Y | Y | Y | Y | Y | Y | N | N | Y | 9/11 |
| Simpson | Y | Y | Y | Y | Y | Y | Y | Y | N | N | Y | 9/11 |
| Hakola | Y | Y | Y | Y | Y | Y | Y | Y | U | U | Y | 9/11 |
| Hyponnen | Y | Y | Y | Y | Y | Y | Y | Y | Y | Y | Y | 11/11 |
| Elhassan | Y | Y | Y | Y | Y | Y | Y | Y | Y | N | Y | 10/11 |
| Lund-Blix | Y | Y | Y | Y | Y | Y | Y | Y | Y | N | Y | 10/11 |
| Brekke | Y | Y | Y | Y | Y | Y | Y | Y | U | U | Y | 9/11 |
| Marjamaki | Y | Y | Y | Y | Y | Y | Y | Y | U | U | Y | 9/11 |

9.2: JBI Critical Appraisal Checklist for Case Control Studies

| Author | 1. Were the groups comparable other than the presence of disease in cases or the absence of disease in controls? | 2. Were cases and controls matched appropriately? | 3. Were the same criteria used for identification of cases and controls? | 4. Was exposure measured in a standard, valid and reliable way? | 5. Was exposure measured in the same way for cases and controls? | 6. Were confounding factors identified? | 7. Were strategies to deal with confounding factors stated? | 8. Were outcomes assessed in a standard, valid and reliable way for cases and controls? | 9. Was the exposure period of interest long enough to be meaningful? | 10. Was appropriate statistical analysis used? | Total |
| --- | --- | --- | --- | --- | --- | --- | --- | --- | --- | --- | --- |
| Adawalla | Y | Y | Y | Y | Y | Y | Y | Y | Y | Y | 10/10 |
| Brikhou | Y | Y | Y | Y | Y | Y | Y | Y | Y | Y | 10/10 |
| Gorham | Y | Y | Y | Y | Y | N | N | Y | Y | Y | 8/10 |
| Stene | Y | Y | Y | Y | Y | Y | Y | Y | Y | Y | 10/10 |
| Tenconi | Y | Y | Y | Y | Y | Y | Y | Y | Y | Y | 10/10 |

9.3: JBI Critical Appraisal Checklist for Randomised Controlled Trials

| Author | 1. Was true randomization used for assignment of participants to treatment groups? | 2. Was allocation to treatment groups concealed? | 3. Were treatment groups similar at the baseline? | 4. Were participants blind to treatment assignment? | 5. Were those delivering treatment blind to treatment assignment? | 6. Were outcomes assessors blind to treatment assignment? | 7. Were treatment groups treated identically other than the intervention of interest? | 8. Was follow up complete and if not, were differences between groups in terms of their follow up adequately described and analyzed? | 9. Were participants analyzed in the groups to which they were randomized? | 10. Were outcomes measured in the same way for treatment groups? | 11. Were outcomes measured in a reliable way? | 12. Was appropriate statistical analysis used? | 13. Was the trial design appropriate, and any deviations from the standard RCT design (individual randomization, parallel groups) accounted for in the conduct and analysis of the trial? | Total |
| --- | --- | --- | --- | --- | --- | --- | --- | --- | --- | --- | --- | --- | --- | --- |
| Gale | Y | Y | Y | Y | Y | Y | Y | Y | Y | Y | Y | Y | Y | 13/13 |

Supplementary Figure 1: Outlier analysis of studies assessing pooled odds ratio of vitamin D supplementation and Type 1 Diabetes Mellitus


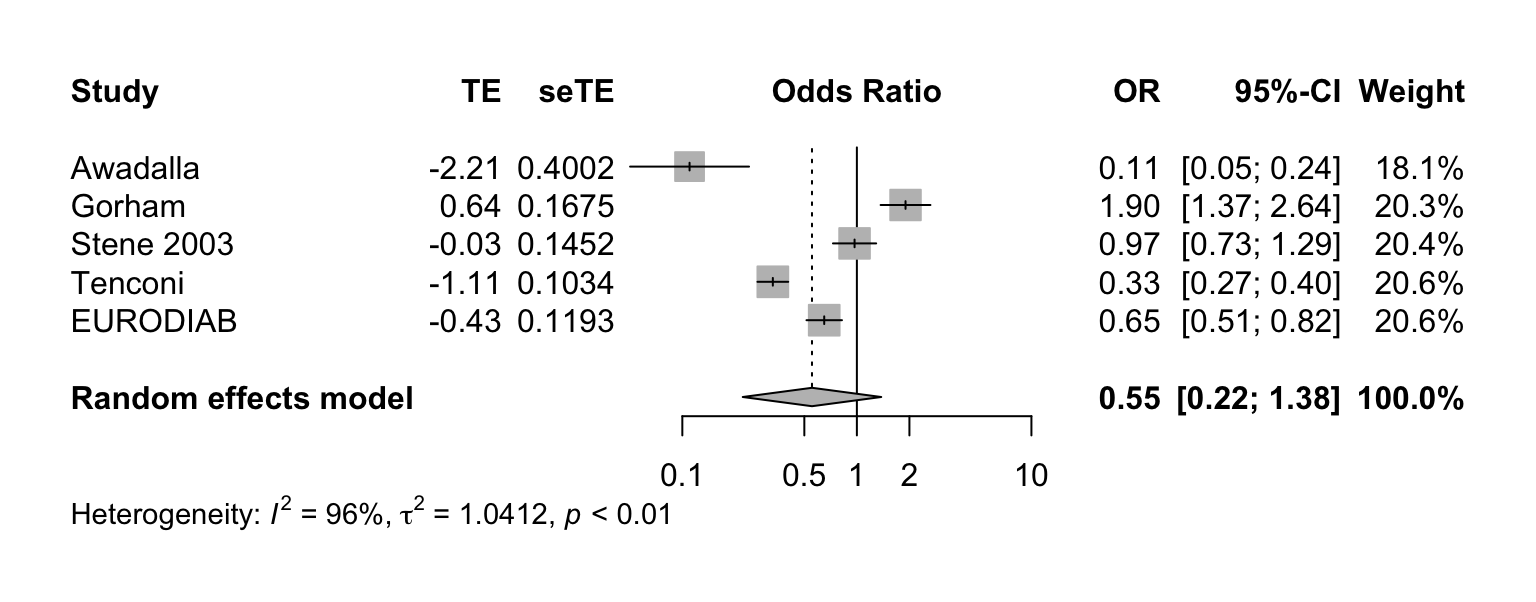


Supplementary Figure 2: Leave-one-out analyses of studies assessing pooled odds ratio of vitamin D supplementation and Type 1 Diabetes Mellitus


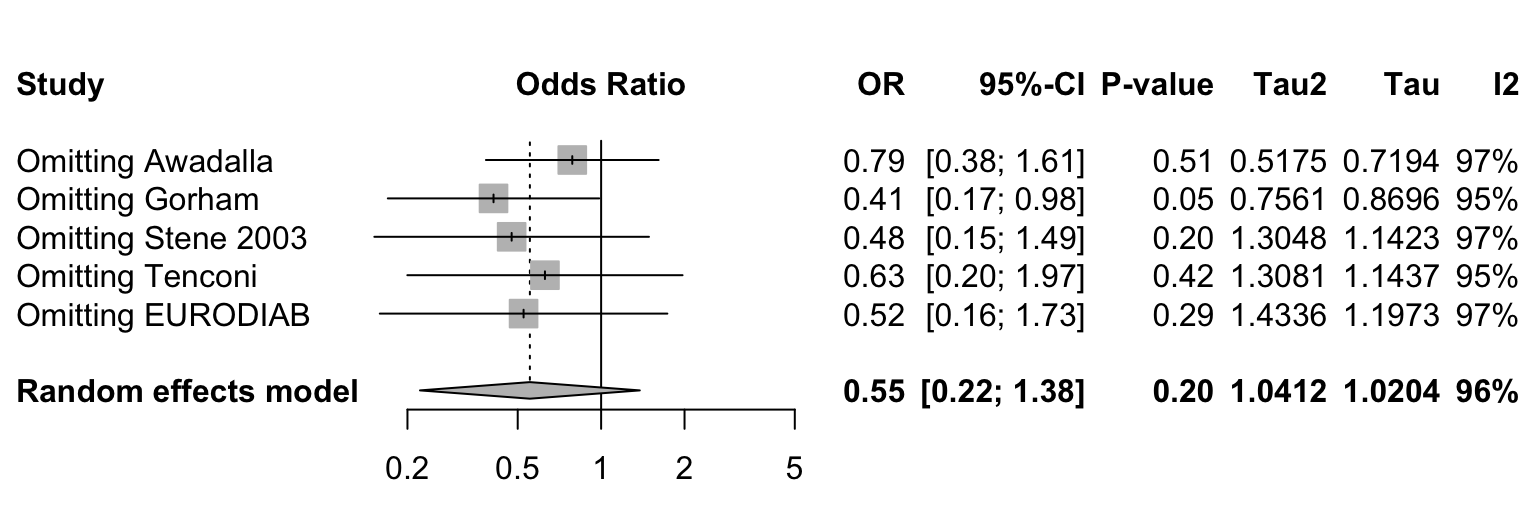


Supplementary Figure 3: Outlier assessment of studies assessing pooled odds ratio of vitamin D supplementation and Islet Autoimmunity


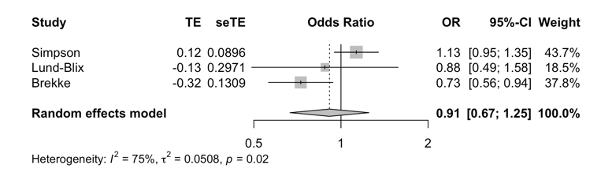


Supplementary Figure 4: Leave-one-out analysis of studies assessing pooled odds ratio of vitamin D supplementation and Islet Autoimmunity


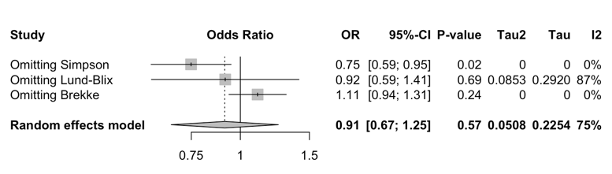

Supplement: Supplementary file 1 [file DataSheet1.docx]
